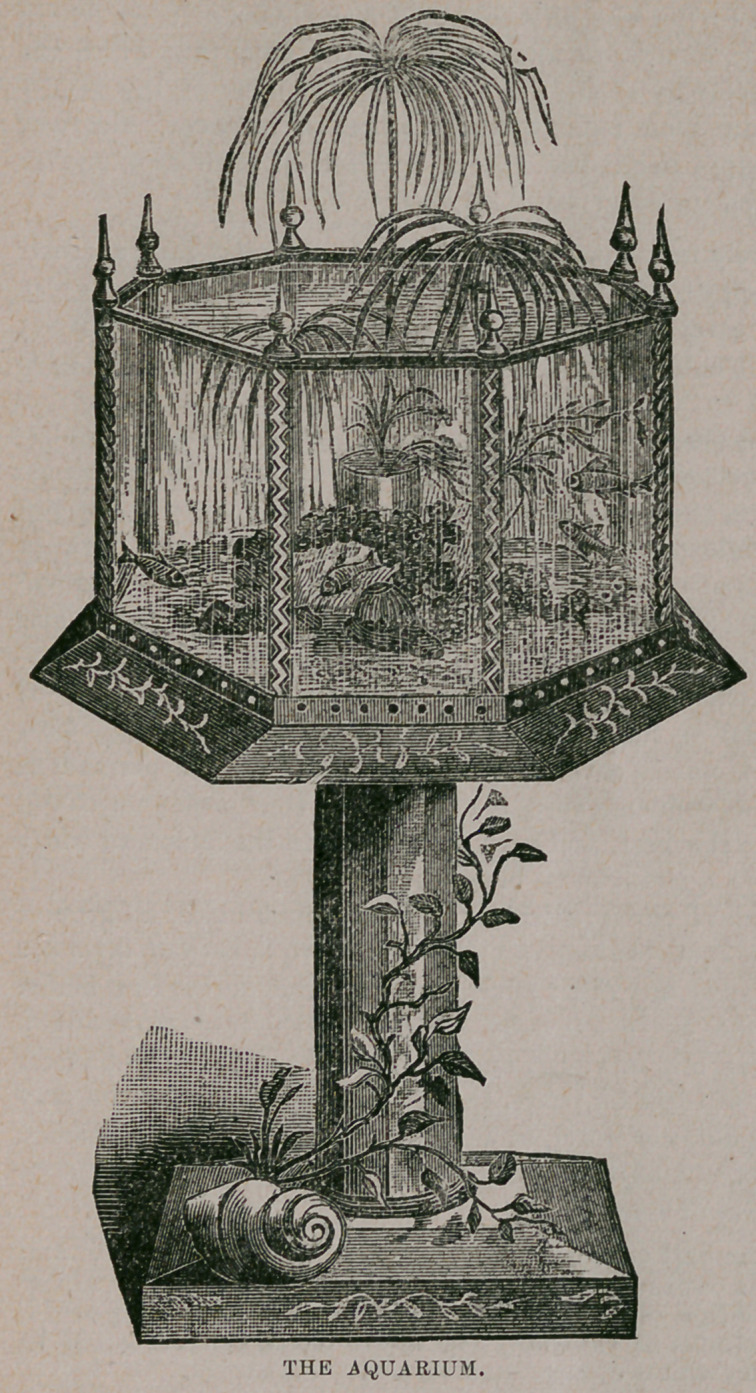# Household

**Published:** 1888-10

**Authors:** 


### HOUSEHOLD.

THE AQUARIUM.—A well stocked aquarium, properly cared for, is a source of never ending enjoyment in the home, and can, by the artistic arrangement of its interior, be made a charming, living picture. Adorn its fine graveled bed with gay hued stones, rare shells, a miniature grotto ; let the leaves of some aquatic plant sway and tremble above them, while the feathery, interlacing roots spread outward, downward, anywhere ; crown the whole with a stately calla lily or umbrella plant ; and as the sun's rays, darting through the vivid green, illuminate the graceful evolutions of the gliding fish, the clear waters enfold a mimic scene of almost fairy-like enchantment.

THE AQUARIUM.

The writer was at one time the possessor of three tanks of various forms and sizes The gold and silver fish were purchases carefully selected on account of brilliant color or peculiar marking, but the other inmates were either the reward of family expeditions or gifts from interested friends.

In my largest aquarium five gold and silver fish, several shiners, three minute bullheads and a few tiny rock bass, found at the bottom of a deep well, dwelt amicably together. Two small turtles and three varieties of snail kept them company. All went merry as a marriage bell until I introduced a stickleback to the assembled crowd. This wee monster proved the quintessence of malicious industry, nibbling the fins and tails of my choicest fish into an uneven fringe in an incredibly short time.

The brilliant, opaline coloring of the sunfish makes them specially attractive, but these ferocious creatures must have a tank to themselves. My fish came, in time, to know me, and although nearly all were shy of strangers, at a tap of my fingers on the glass they would sally from their hiding places and cluster together. Sometimes I fancied that even the snails recognized me ; be that as it may, the intelligence of my hardshell turtles was an undoubted fact. One of them would manage by adroit climbing to somersault himself out of the aquarium on to the floor, where he strayed contentedly about, until a certain member of the family entering he would follow him from room to room, wherever he went. Six soft shell turtles from the Mississippi banks had special quarters of their own, and as genuine curiosities, were often the observed of all observers.

A Rose Jar.—Comparatively few housekeepers rate these useful jars at their proper value. If you are happy in the possession of one, it should be opened every morning for an hour, then carefully closed. All your friends will ask, "What gives your room so delightful a fragrance ?" It is such a pure yet delicious odor that it charms everyone.

The preparation of the rose stock should be detailed to the care taking member of the family, who never forgets anything. Gather the rose petals in the morning; let them stand in a cool place, toss them up lightly for one hour to dry ; then put them in layers, with salt sprinkled over each layer, into a large covered dish—a glass butter dish is a convenient receptacle. You can add to this for several mornings, till you have enough stock—from one pint to a quart, according to the size of the jar ; stir every morning and let the whole stand for ten days. Then transfer it to a glass fruit jar, in the bottom of which you have placed two ounces of allspice, coarsely ground, and as much stick cinnamon, broken coarsely. This may now stand for six weeks, closely covered, when it is ready for the permanent jar, which may be as pretty as your ingenuity can devise or your means purchase. Those with double covers are the best, and very pretty ones in the blue and white Japanese ware, holding over a quart, can be bought for a dollar.

Have ready one ounce each of cloves, allspice, cinnamon and mace, all ground (not fine) ; one ounce of orris root, bruised and shredded ; two ounces of lavender flowers, and a small quantity of any other sweet scented dried flowers or herbs. Mix together, and put into the jar in alternate layers with the rose stock, and a few drops of oil of rose, geranium, or violet, and pour over the whole one quarter pint of good cologne. This will last for years, though from time to time you may add a little lavender or orange-flower water, or any nice perfume, and some seasons a few fresh rose petals. You will derive a satisfaction from the labor only to be estimated by the happy owners of similar jars.

White Cake.—One and one-half cups of sugar, one-half cup each of butter and corn starch, one and one-half cups of flour, one-half cup of sweet milk, the whites of six eggs, and two teaspoonfuls of baking powder ; flavor with one teaspoonful of lemon and one-half teaspoonful of vanilla. The above recipe can be varied by using two whole eggs and the whites of two, making a very rich color.

Ham and Eggs.—Take six eggs and three tablespoonfuls of ham chopped very fine. Beat the eggs, and, after melting a lump of butter in the frying pan, drop the eggs into it and stir the ham in. The ham has, of course, been cooked, either fried or boiled. Season with pepper. This is a good way to use up pieces of meat that are left from dinner.

## Figures and Tables

**Figure f1:**